# Supplementary material for: Exploring the Link between Work Addiction Risk and Health-Related Outcomes Using Job-Demand-Control Model
Source: Int J Environ Res Public Health. 2020 Oct 19;17(20):7594. doi: 10.3390/ijerph17207594 (PMC7593928; doi:10.3390/ijerph17207594)

Table S1. Occupations and education levels across the Karasek quadrants.

Variables	Job-Demand-Control model of Karasek				p-value
	**Active**	**High-strain**	**Low-strain**	**Passive**	
**Education level**					
General Certificate of Secondary Education	0.0%	4.6%	0.0%	0.0%	
A-level	3.4%	0.0%	2.7%	20.0%	
Higher National Diploma	5.7%	9.1%	5.4%	26.7%	**.0002**
Bachelor degree	8.0%	18.2%	16.2%	20.0%	
Master degree	83.0%	68.2%	75.7%	33.3%	
**Occupational group**, n (%)					
Merchants – Business	5.7%	0.0%	2.7%	0.0%	
Employees	8.0%	36.4%	13.5%	40.0%	**.050**
Intermediate profession	6.8%	9.1%	2.7%	6.7%	
Inactive employment	3.4%	0.0%	13.5%	13.3%	
Manager- Intellectual profession	76.1%	54.6%	65.6%	40.0%	

Figure S1_NumberOfSubjectsRequired_300dpi

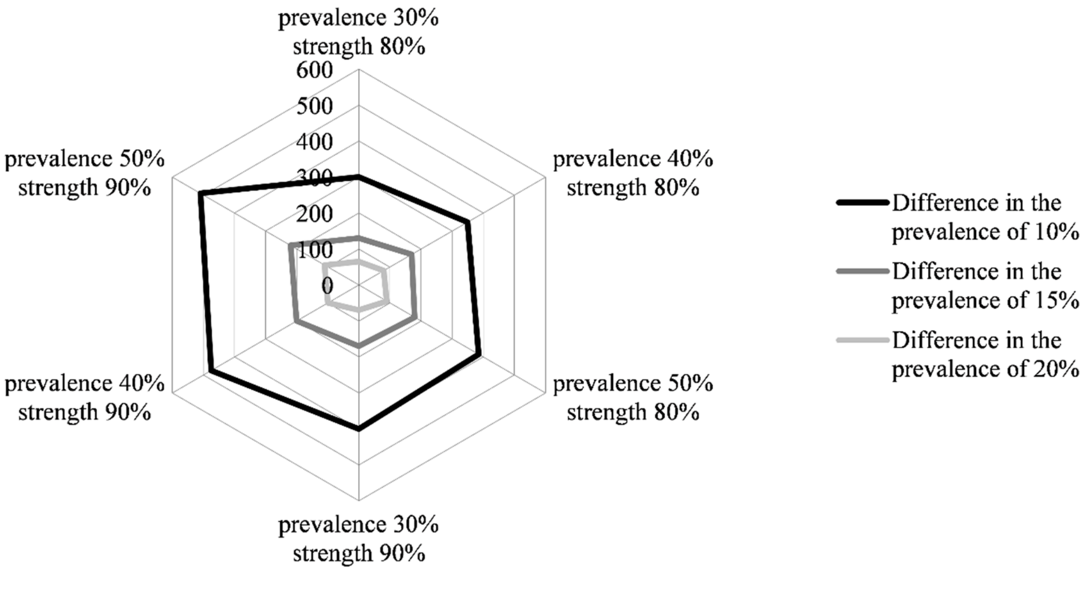

Supplement: Supplementary file 1 [file ijerph-17-07594-s001.pdf]
